# Efficacy of peat-based bioformulation of microbial co-inoculants with silicon for growth promotion of rubber plants

**DOI:** 10.1371/journal.pone.0331899

**Published:** 2025-10-08

**Authors:** Hao Ling, Feng Xu, Imran Shabbir, Zulkefly Sulaiman, Muhammad Shahbaz, Dunia A. Al Farraj

**Affiliations:** 1 Sino-thai International College, Assumption University of Thailand, Bangkok, Thailand; 2 China Merchants Chongqing Communications Technology Research & Design Institute Co., Ltd., Chongqing, China; 3 Department of Land Management, Faculty of Agriculture, Universiti Putra Malaysia, Serdang, Selangor, Malaysia; 4 Institute of Agronomy, Bahauddin Zakariya University, Multan, Punjab, Pakistan; 5 Department of Crop Science, Faculty of Agriculture, Universiti Putra Malaysia, Serdang, Selangor, Malaysia; 6 Institute of Plantation Studies, Universiti Putra Malaysia, Serdang, Selangor, Malaysia; 7 Department of Botany and Microbiology, College of Science, King Saud University, Riyadh, Saudi Arabia; Graphic Era Institute of Technology: Graphic Era Deemed to be University, INDIA

## Abstract

Recently, microbial consortia of rhizobacteria and arbuscular mycorrhizal fungi (AMF) had demonstrated the potential as plant growth promoting microbes in sustainable agriculture. This study aimed to investigate the effect of a peat moss-based formulation of *Enterobacter* sp. UPMSSB7, *Glomus mosseae*, and silicon (Si) on the survival of microbial inoculants under storage conditions for 24 weeks. The study further assessed the potential of this bioformulation to promote the growth of rubber plants in a glasshouse trial. The *Enterobacter* sp. UPMSSB7 isolated from rubber tree’s rhizosphere, can solubilize silicates and has plant growth promoting properties. *G. mosseae* is an AMF, having symbiotic relationship with majority of cultivated crops. The application of Si has emerged as a sustainable strategy for crop health. It improves soil fertility through nutrient maintenance and also alleviates various biotic and abiotic stresses. Results from laboratory test revealed that bioformulation of co-inoculants with Si sustained a high survivability of *Enterobacter* sp. (18 × 10^8^ CFU g^-1^) and *G. mosseae* (35 spores per 10 g) in formulation for up to 24 weeks of storage. Results from the glasshouse experiment revealed that 24 weeks after treatment with bioformulation of co-inoculants with Si increased the stem height, girth, leaf area, dry weight of shoot and root, chlorophyll content, microbial population of *Enterobacter* sp. (1.4 × 10^8^ CFU g^-1^ soil) and *G. mosseae* (78 spores/10 g soil) in rhizosphere and also increased N, P, K and Si contents in rubber seedlings than bioformulation of single inoculant with Si and control. Our findings indicate that peat moss-based formulation of co-inoculants *Enterobacter* sp. UPMSSB7 and *G. mosseae* added with Si proved to be the most effective. This formulation not only maintained good microbial survivability but also significantly enhanced the rubber plants growth compared to the bioformulation of single inoculants. This promising approach using a peat moss-based formulation of microbial co-inoculants with Si, could be further explored for growth enhancement of rubber trees under field conditions.

## 1. Introduction

*Hevea brasiliensis* Muell. Arg., commonly known as the rubber tree, is cultivated for the production of natural rubber [1]. The rubber industry holds significant economic importance in both industrialized and emerging nations, supplying its products to numerous economic and industrial sectors [2]. Malaysia is the fifth-largest natural rubber producer globally [3]. The intensive use of high-dose fertilizers in rubber tree plantations not only diminishes beneficial soil microorganisms [4], but also imposes considerable environmental and economic burdens [5]. Continuous application of chemical fertilizers for a long period of time can decrease soil pH, which is closely linked to a reduction in microbial population [6]. Moreover, use of pesticides has also been increased in agriculture which results in reduction in microbes by degrading microbial structure, biochemical reactions and cellular processes [7]. The monocropping of rubber trees has increased a variety of adverse environmental effects, such as depletion of soil nutrients, reduction in beneficial microbe activities, and soil degradation [8].

Silicon (Si) is a beneficial element known for increased nutrient availability [9]. Promoting Si among farmers has prospects for sustainable agriculture by crop improvement [10]. Plants can uptake Si as monosilicic acid which is a soluble form that promotes plants growth [11]. The Si is abundant in soil however, it is bound to other minerals and make insoluble silicates that are converted into soluble silicates by the action of microbes [12]. There are many different microbes present in soil, however only a few can dissolve insoluble silicates. There are silicate solubilizing bacteria known as plant growth-promoting rhizobacteria (PGPR) that can solubilize the insoluble silicates. For example, *Enterobacter ludwigii* GAK2 solubilized the silicates on glucose agar medium and significantly increased Si content in rice plant tissues under pot experiment [13]. The *Enterobacter* sp. improved the growth of both sunflower [14] and maize plants [15]. PGPR improve the crops quality by reducing the use of chemical fertilizer and enhancing the nutrient uptake [16]. PGPR can mitigate the adverse impacts of chemical fertilizers [17] and actively modulate plant physiology by encouraging the synthesis of hormones, enhancing growth, and improving nutrient yields [18].

The arbuscular mycorrhizal fungi (AMF) are known as biostimulants, biocontrol and bioenhancers [19]. The AMF can improve the nutritional quality of crops by producing carotenoids and specific volatile compounds [20]. Emmanuel and Babalola [21] observed elevated levels of sugars, minerals and organic acids due to application of AMF, leading to enhanced quality in citrus fruit. The AMF stimulates increased accumulation of mineral nutrients, total soluble phenolics, chlorophyll, carotenoids, and anthocyanins [22]. The application of *Glomus mosseae* increased the root and shoot dry weights of rubber plants [23]. The excessive application of inorganic fertilizers reduced colonization of AMF [24]. The use of different microbial inoculants provides many benefits than a single microbial inoculant [25]. Such as, consortium of fungi and bacteria proved to be effective than recommended fertilizer for rubber plants [26]. The *Bacillus megaterium* and AMF (*Funneliformis mosseae*) as co-inoculants improved the root morphology, soil nutrient availability, and plant growth of *Elymus nutans* Griseb [27].

Microbial bioformulations are widely used to boost plant growth and provide protection [28]. The effectiveness of these microbial strains depends on their ability to adapt to the new environment and compete with native microbes [29]. Carrier formulations are used to retain the viability of microbes during long storage periods [30]. Hence, microbes are used with the suitable carriers to maintain viability of inoculants and to be effective for plant growth promotion.

The stability of a bioformulation is expected to remain effective for a period of six months to a year [31]. Stability testing involves conducting serial dilution plating and microbial population density should not be less than 10^4^ CFU g^-1^ sample [32]. Bioformulations are of different types such as liquid, solid, metabolites, and encapsulated [33]. Solid formulations provide a protective, nutrient-rich environment for microbial inoculants while enhancing storage efficiency [34]. The AMF inoculum could be stored in vermicule formulation for five months under temperature range of 20°C to 30ºC and maintained the maximum AMF spore count (5–6/100g) [35]. Bioformulation is a carrier-based system that enhances microbial survival for an extended period [30]. Some commonly used carrier materials, such as peat, serve as mediums for microbial inoculants [36]. Peat moss is a carrier material most often used for *Glomus* sp. storage [37]. Peat moss which contains high nitrogen content and high availability of labile carbon was the best for *Enterobacter cloacae* UW5 survival after 4 weeks [38]. *Enterobacter* spp. are among few of the bacteria, while *Glomus* sp. are among few of the fungi that have been widely used as inoculants in formulations [39–41].

Although, there have been many studies conducted to develop a bioformulation of a single inoculant to evaluate its storage stability and efficacy for plant growth promotion however, there have been no attempts to develop a bioformulation consisting of *Enterobacter* sp., *G. mosseae* and Si to evaluate its viability and stability in storage and then to evaluate the stored bioformulation for growth promotion of rubber plants. Therefore, a laboratory experiment was conducted to develop a peat moss-based formulation of *Enterobacter* sp. UPMSSB7, *G. mosseae* and Si to evaluate its viability in storage for 24 weeks. The study further assessed the efficacy of this stored bioformulation to promote the growth of rubber seedlings in a glasshouse trial.

## 2. Materials and methods

### 2.1. Microbial inoculants

No permits or approvals were required for this work as no protected species were collected for this study. In our previous study, bacterial isolate UPMSSB7 was isolated from the rhizosphere of rubber plants and identified as *Enterobacter* sp. based on partial sequencing of 16S rRNA by PCR using universal forward (5´- GAGTTTGATCCTGCTCAG-3´) and reverse (5´-GTTACCTTGTTACGACTT-3´) primers (BioSune Biotechnology Co. Ltd., China) [42]. This isolate was grown in Luria–Bertani (LB) broth medium and then incubated at 25 °C for 48 h on a rotary shaker at 200 rpm. Afterwards, the suspension of the isolate that exceeded 1 × 10^8^ CFU mL^-1^ was used to prepare bioformulation [43]. The AMF inoculum of *Glomus mosseae* (UK118) was sourced from the International Collection of Vesicular Arbuscular Mycorrhizal Fungi (INVAM), USA. The inoculum was then propagated on maize for 10 weeks glasshouse pot culture using sterilized sand. As a source of insoluble Si, calcium silicate (HmbG^®^ Chemicals, Hamburg, Germany) was utilized.

### 2.2. Development of peat-based bioformulation for storage under laboratory trial

#### 2.2.1. Preparation of bioformulation and storage conditions.

The bioformulation was prepared with a slight modification as per method described by Wu *et al.* [44]. In this study, we used commercially available peat moss (Agroniche Pvt. Ltd., Holland). Its pH was 4.6, and its chemical composition was as follows: 1) carbon (51.3% in peat), oxygen (41.1%), hydrogen (6.08%), nitrogen (1.19%), and sulfur (0.16%). Peat moss (500 g) was filled in a polyethylene bag (20 cm x 30 cm) and sterilized by using autoclave at a temperature of 121 ˚C for 3 h (1 h intervals). Under sterile conditions, sterilized peat in each polyethylene bag was added with LB broth (250 mL) and suspension of *Enterobacter* sp. UPMSSB7 (1 × 10^8^ CFU mL^-1^) (20 mL). This mixture was then air-dried in a laminar airflow cabinet until it reached a workable moisture level of 15–20% [45]. Thereafter, the spore density of previously prepared AMF inoculum was calculated by a wet-sieving and decanting method [46]. The AMF inoculum, containing at least 20 spores per gram of dry sand, was chosen for incorporation into the bioformulation. The AMF inoculum (20 spores/g of dry sand) at the rate of 100 g and Si at 4 g were added in each polyethylene bag. These bags were sealed and preserved at room temperature for 24 weeks of storage.

#### 2.2.2. Experimental design and treatments.

This laboratory experiment was carried out in a completely randomized design (CRD) with three replicas. The following treatments were included: T1 (Control), sterile peat applied with no microbial inoculant; T2 (AMF + Si), formulation of AMF (*G. mosseae*) with Si; T3 (Eb + Si), formulation of *Enterobacter* sp. with Si; while T4 (Eb + AMF + Si), formulation of *Enterobacter* sp., *G. mosseae* and Si.

#### 2.2.3 Survival of microbial inoculants in the formulation.

The spore density of *G. mosseae* and population of *Enterobacter* sp. in peat formulation was determined at 4-week intervals up to 24 weeks of storage. Formulation inoculum (10 g) was diluted in sterile distilled water (90 mL) and serially diluted. The *G. mosseae* spore density in the formulation was calculated by a wet-sieving and decanting procedure [46]. The *Enterobacter* sp. population was calculated on glucose agar modified with magnesium trisilicate (0.25%) using serial dilution [47].

### 2.3. Evaluation of bioformulation in a glasshouse experiment

#### 2.3.1. Experimental design and treatments.

This glasshouse study was carried out in randomized complete block design (RCBD) with five replicas and two plants per replica. The treatments involved: T1 (Control): sterile peat was applied; T2 (Si): sterile peat containing Si (4 g) was applied; T3 (AMF): peat-based bioformulation containing AMF was applied; T4 (AMF + Si): peat-based bioformulation containing AMF with Si (4 g) was applied; T5 (Eb): formulation containing *Enterobacter* sp. was applied; T6 (Eb + Si): formulation containing *Enterobacter* sp. with Si was applied; T7 (Split AMF + Eb + Si): formulation of AMF alone was applied initially at start and then, a week later *Enterobacter* sp. and Si were applied; T8 (Consortium of AMF + Eb + Si): formulation of *Enterobacter* sp., AMF with Si, were applied altogether. This study was conducted over 24 weeks period after the formulation treatments were applied. The tap water was applied daily at 300 ml per polybag for 24 weeks. The fertilizer, known as RISDA 1 (N-P-K-Mg = 10.7-16.6-9.5-2.4) was applied twice at 75 g per plant for each dose. The plants in the control treatment (T1) received a total dose of recommended fertilizer, while all other treatments (T2, T3, T4, and T5) received 70% recommended fertilizer.

#### 2.3.2. Plant materials and bioformulation.

For glasshouse study, the rubber seedlings (2 months old) with 2 whorl leaves of PB-350 clone were used. Before formulation application, plants were transferred from old to new polybags (40 cm x 40 cm). Each polybag contains 15 kg of autoclaved soil and a single seedling. Soil used in this study is considered as one of the best soils for rubber trees and categorized as Munchong soil series [48]. For the application of peat-based formulation to rubber plants, soil was carefully removed around the roots without damaging them. For glasshouse experiment, each formulation was prepared as per as described previously in the development of formulation section. The peat-based bioformulation, stored at room temperature for 24 weeks, was used in this glasshouse study.

It was inoculated at 500 g per rubber plant in a circular furrow around roots and then covered with soil. The *Enterobacter* sp. UPMSSB7 inoculum was prepared as per described previously in preparation of bioformulation by mixing B broth (250 mL) and suspension of *Enterobacter* sp. (20 mL). The *Enterobacter* sp. population in the bioformulation was at 1 × 10^8^ CFU g^-1^. The AMF inoculum was added in the bioformulation at the rate of 100 g. The AMF spores were at least 20 spores g^-1^ at the time of use.

#### 2.3.3. Plant growth and nutrient contents analysis.

After 24 weeks, the stem height of plants was measured using measuring ruler, while girth size using vernier caliper. The chlorophyll meter (SPAD-502, Minolta Osaka, Japan) was utilized to record the total chlorophyll content, while leaf area meter machine (Li-Color LI-3100^©^ Area meter) was used for leaf area. Tap water was used to wash the fresh roots and root analysis was done by an EPSON WhinRhizo root scanner to obtain root length, root surface area and root volume parameters. The leaf nutrient contents such as, N, P and K, were determined using a method described by Rubber Research Institute, Malaysia [49]. The autoclaved induced digestion technique was applied to quantify Si content in rubber plant’s root and shoot [50].

#### 2.3.4. Spore density and root colonization of *G. mosseae* and population of *Enterobacter* sp.

After 24 weeks of bioformulation application, soil samples were extracted from the rhizosphere of each treatment to determine the *G*. *mosseae* spore density. The wet-sieving and decanting method was applied to calculate spore density, which was represented as the number of *G*. *mosseae* spores per 10 g of soil [46]. Fine lateral root samples of plants were collected 24 weeks after bioformulation application to assess the mycorrhizae colonization. The root colonization by *G*. *mosseae* was assessed using a procedure outlined by McGonigle et al. [51]. First, freshly cut roots were cleaned with deionized water. Randomly selected fine lateral roots (2 g) were chopped into 1–2 cm segments, and then added to a 25 mL MacCartney bottle. The segments were then heated in a water bath for one hour at 80 °C and soaked in a 10% KOH solution for three days (changing the solution after 24 hours). After that, the segments were cleaned with distilled water and stained using trypan blue (0.05%) in lacto-glycerol (a solution of equal parts water, lactic acid, and glycerin). The presence of arbuscules, vesicles, and hyphae of mycorrhizae was observed under a microscope (Leica DM5000B, Wetzlar, Germany) to aid in measuring these segments. The population of *Enterobacter* sp. was esimated on glucose agar containing magnesium trisilicate (0.25%) using serial dilution [47].

### 2.4. Statistical analysis

The data for all parameters collected from each experiment were analyzed by one-way ANOVA using PROC ANOVA/GLM and treatments means were separated by Least Significant Difference (LSD) test at *P ≤ *0.05 using SAS version 9.4 (SAS Inc., USA).

## 3. Results

### 3.1. Development of peat-based bioformulation for storage

#### 3.1.1. Survivability of microbial inoculants in bioformulation during storage.

**Fig 1:**
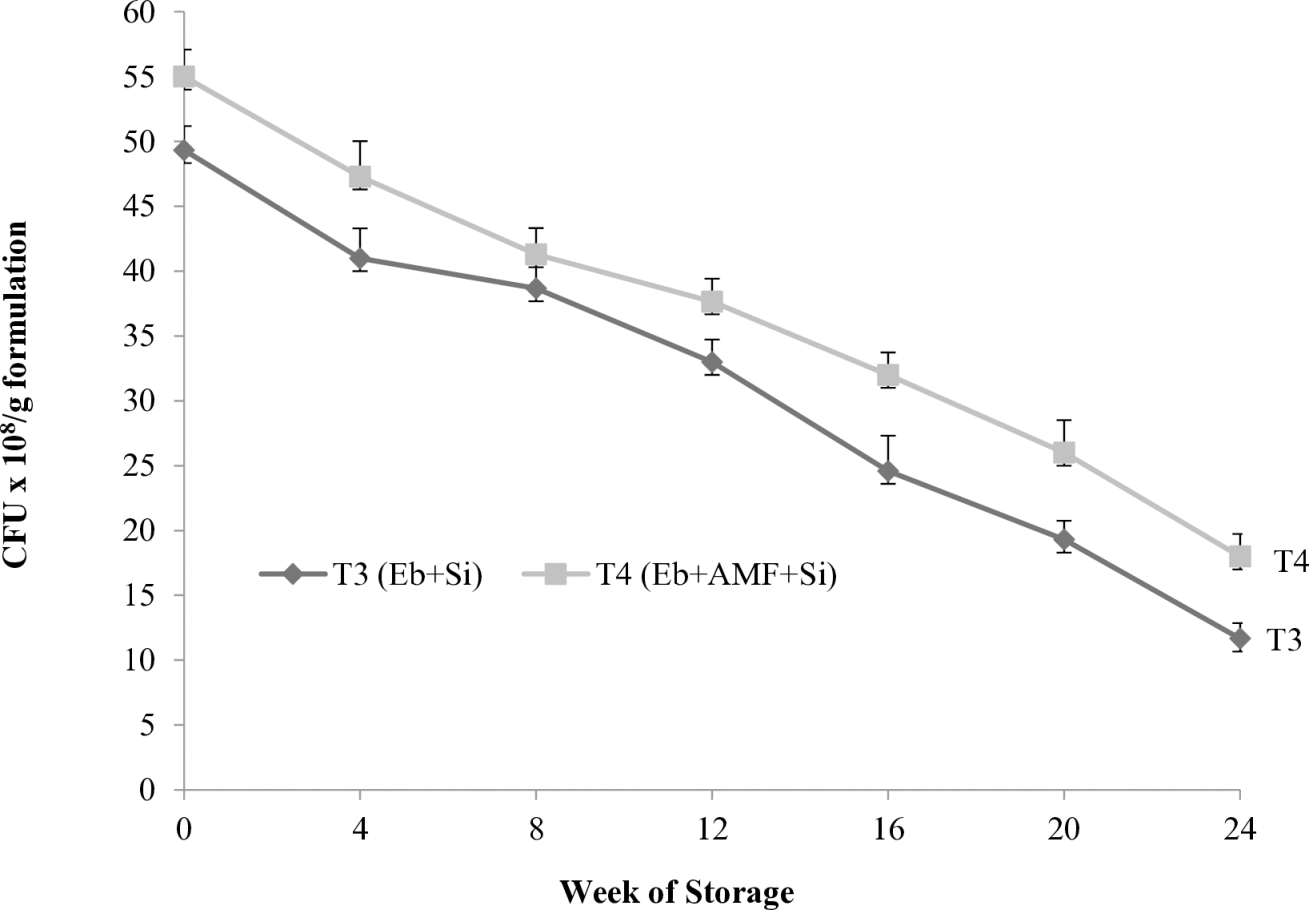
Survivability of *Enterobacter* sp. **UPMSSB7 in bioformulation of microbial agents with Si during 24 weeks of storage.** Note: Si, Eb and AMF used were calcium silicate, *Enterobacter* sp. UPMSSB7 and *G. mosseae*, respectively. Values represent the means of three replication with vertical bars are standard error (SE). Figs 1 and 2 share the same legend.

**Fig 2:**
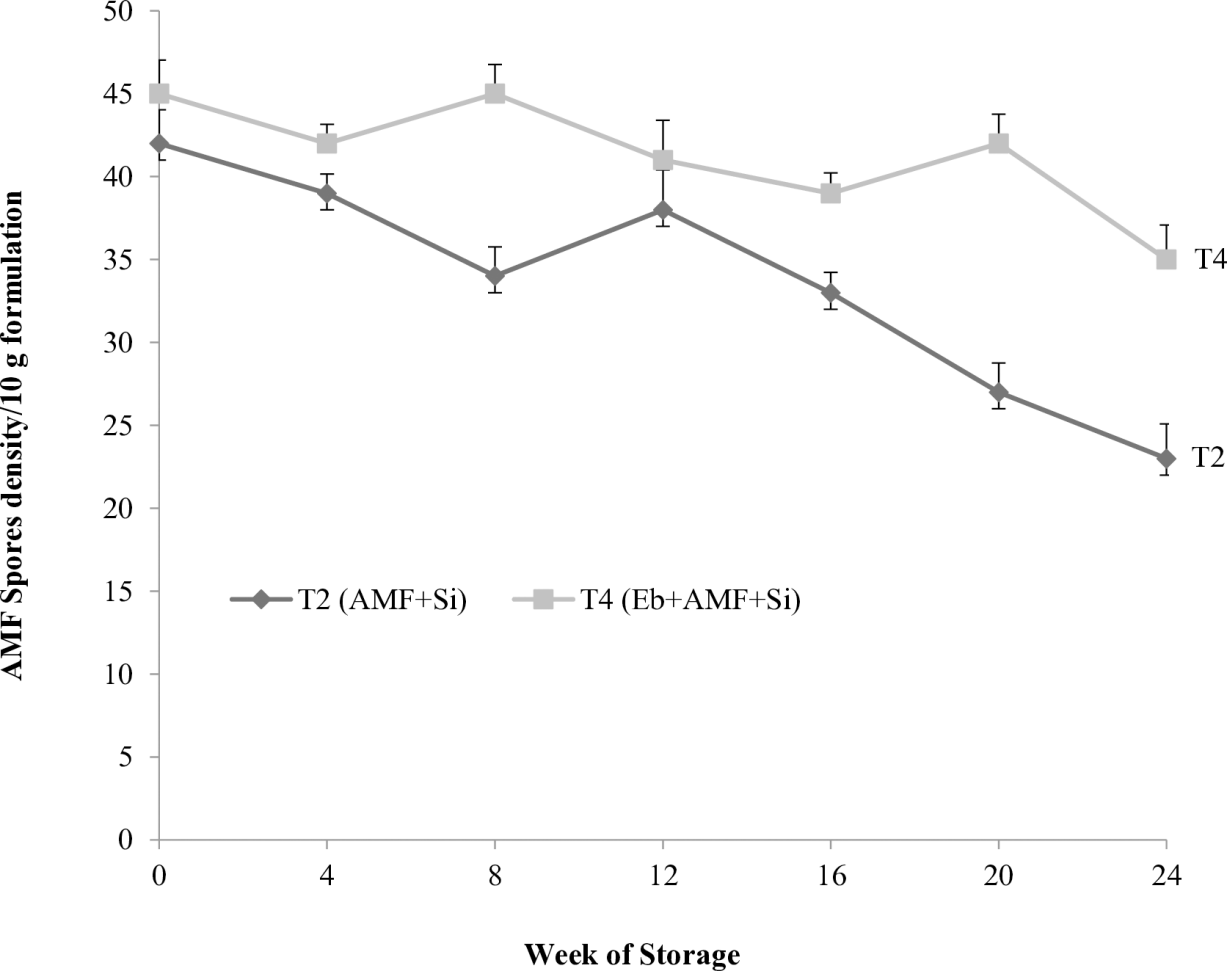
Survivability of *G. mosseae* spore density in bioformulation of microbial agents with Si during 24 weeks of storage.

The results revealed that *Enterobacter* sp. was capable of surviving in peat-based bioformulations (Fig 1). The T3 (Eb + Si) and T4 (Eb + AMF + Si) formulations showed the ability to maintain bacterial population above 10^8^ CFU g^-1^ during 24 weeks of storage. This is one of the desirable properties required in bioformulations. After 24 weeks of storage, T4 formulation showed the highest bacterial survivability (18 x 10^8^ cfu g^-1^), while T3 recorded lower survivability (11.7 x 10^8^ cfu g^-1^). The results showed that there was a fluctuating trend of AMF spore density during storage (Fig 2). During 24 weeks of storage, T4 formulation maintained a stable spore density with a minor decrease, while it was drastically reduced in T2 formulation. After 24 weeks of storage, T4 formulation recorded the highest spore density of AMF (35 spores per 10 g), followed by T3 (23 spores per 10 g), with a significant difference between them. The *Enterobacter* sp. population and AMF spores were not detected in other formulations that were prepared without *Enterobacter* sp. and AMF inoculum.

### 3.2. Evaluation of bioformulation in a glasshouse experiment

#### 3.2.1. Plant growth performance.

The growth parameters were significantly increased with bioformulation of both microbial inoculants compared to bioformulation of alone inoculants and control (Tables 1 and 2 and S1 Fig). After 24 weeks inoculation with bioformulation, both T7 and T8 had significantly (*P* < 0.01) enhanced the growth parameters than other treatments. However, there was no significant difference between T7 and T8. The T5 and T6 increased the growth parameters than T1, T2, T3 and T4. The T1 and T2 treatments had the lower measured growth parameters than other treatments (Tables 1 and 2).

**Table 1:** The growth performance of rubber plants affected by bioformulation in a glasshouse assay 24 weeks after inoculation.

Bioformulation treatment	Stem height (cm)	Girth size (mm)	Leaf area (cm^2^)	Chlorophyll content (SPAD value)	Dry weight (g plant^−1^)	
					Root	Shoot
T1 (Control)	72 ± 1.24 d	5.27 ± 0.23 g	2110 ± 50 d	40.32 ± 1.45 de	13.8 ± 1.12 e	12.1 ± 0.71 d
T2 (Si)	69 ± 3.28 d	5.71 ± 0.34 fg	2267 ± 95 c	38.71 ± 2.01 e	15.9 ± 0.55 de	13.7 ± 1.11 cd
T3 (AMF)	81 ± 4.34 c	6.7 ± 0.48 de	2411 ± 145 b	45.46 ± 2.95 cd	18.7 ± 1.01 cd	15.4 ± 1.04 cd
T4 (AMF + Si)	82 ± 1.43 c	6.39 ± 0.15 ef	2441 ± 57 c	45.48 ± 2.55 cd	18.6 ± 0.86 cd	16.7 ± 0.89 c
T5 (Eb)	91 ± 5.89 b	7.49 ± 0.6 cd	2708 ± 182 a	50.73 ± 3.65 bc	21.2 ± 0.97 bc	20.6 ± 1.49 b
T6 (Eb + Si)	92 ± 1.28 b	7.64 ± 0.35 bc	3017 ± 39 b	50.52 ± 1.17 c	22.7 ± 2.06 b	22.2 ± 0.75 b
T7 (Split AMF + Eb + Si)	103 ± 3.84 a	8.44 ± 0.23 ab	3312 ± 105 a	56.52 ± 1.05 ab	27.0 ± 1.16 a	27.7 ± 2.01 a
T8 (Consortium of Eb + AMF + Si)	108 ± 3.35 a	8.82 ± 0.17 a	3514 ± 59 a	59.90 ± 0.93 a	29.1 ± 1.72 a	29.2 ± 2.29 a

Note: T1 (Control) = sterilized peat applied; T2 (Si): sterile peat containing Si (4 g) was applied; T3 (AMF): peat-based bioformulation containing AMF was applied; T4 (AMF + Si): peat-based bioformulation containing AMF with Si (4 g) was applied; T5 (Eb): formulation containing *Enterobacter* sp. was applied; T6 (Eb + Si): formulation containing *Enterobacter* sp. with Si was applied; T7 (Split AMF + Eb + Si): formulation of AMF alone was applied initially at start and then, a week later *Enterobacter* sp. and Si were applied; T8 (Consortium of AMF + Eb + Si): formulation of *Enterobacter* sp., AMF and Si, were applied altogether.

**Table 2:** The root growth performance of rubber plants affected by the bioformulation in a glasshouse assay 24 weeks after inoculation.

Bioformulation treatment	Root length (cm)	Root surface area (cm^2^)	Root volume (cm^3^)
T1 (Control)	594 ± 32.51 e	274 ± 18.97 e	11.89 ± 0.51 e
T2 (Si)	602 ± 29.46 e	302 ± 14.04 de	12.73 ± 0.82 de
T3 (AMF)	816 ± 41.14 d	328 ± 16.5 cd	15.54 ± 0.34 cd
T4 (AMF + Si)	804 ± 37.50 d	329 ± 20.78 cd	16.07 ± 1.26 c
T5 (Eb)	1059 ± 61.91 b	350 ± 21.6 bc	19.41 ± 0.85 b
T6 (Eb + Si)	1150 ± 31.12 b	376 ± 12.82 b	21.36 ± 0.89 b
T7 (Split AMF + Eb + Si)	1526 ± 26.26 a	432 ± 14.42 a	26.06 ± 1.77 a
T8 (Consortium of Eb + AMF + Si)	1617 ± 21.14 a	460 ± 16.18 a	28.01 ± 1.15 a

#### 3.2.2. Plant nutrient contents analysis.

After 24 weeks, plants treated with bioformulation of both microbial inoculants had increased (*P* < 0.01) nutrient contents compared to bioformulation of alone inoculants and control (Table 3). After 24 weeks, nutrient contents (N, P and K) were increased in T7 and T8 than other treatments. However, there was no significant difference between T7 and T8. Plants of T5 and T6 had increased N, P and K contents than plants of T1, T2, T3 and T4. The Si content in root and shoot also increased (*P* < 0.01) in T7 and T8 than other treatments. However, there was no significant difference between T7 and T8. The T5 and T6 had increased Si content in both the root and shoot than T1, T2, T3 and T4. The plants of T1 and T2 exhibited lower Si content in both the root as well as shoot than other treatments (Table 3).

**Table 3:** The nutrient (N, P, K and Si) contents of rubber plants affected by bioformulation in a glasshouse assay 24 weeks after inoculation.

Bioformulation treatment	Leaf nutrient contents (% of dry weight)			Si content (g kg^-1^ of dry weight)	
	N	P	K	Shoot	Root
T1 (Control)	1.78 ± 0.17 e	0.061 ± 0.009 d	0.61 ± 0.08 d	5.36 ± 0.46 d	3.11 ± 0.40 d
T2 (Si)	1.87 ± 0.16 de	0.073 ± 0.008 cd	0.61 ± 0.05 d	6.07 ± 0.67 d	3.64 ± 0.22 d
T3 (AMF)	2.35 ± 0.11 cd	0.093 ± 0.014 cd	0.78 ± 0.07 c	11.45 ± 0.83 c	6.22 ± 0.70 c
T4 (AMF + Si)	2.39 ± 0.25 cd	0.106 ± 0.014 c	0.84 ± 0.05 c	11.54 ± 0.88 c	6.74 ± 0.62 c
T5 (Eb)	2.65 ± 0.16 bc	0.146 ± 0.011 b	0.98 ± 0.07 b	18.03 ± 0.58 b	9.31 ± 0.48 b
T6 (Eb + Si)	3.03 ± 0.03 b	0.153 ± 0.012 b	1.01 ± 0.01 b	19.08 ± 3.00 b	11.33 ± 0.75 b
T7 (Split AMF + Eb + Si)	4.12 ± 0.27 a	0.201 ± 0.01 a	1.15 ± 0.02 a	26.76 ± 2.83 a	16.19 ± 0.90 a
T8 (Consortium of Eb + AMF + Si)	4.15 ± 0.34 a	0.233 ± 0.017 a	1.23 ± 0.01 a	29.24 ± 2.04 a	18.4 ± 1.63 a

#### 3.2.3. G. mosseae spore density and root colonization, as well as Enterobacter sp. population.

The spore density was significantly higher (*P* < 0.01) in bioformulation treatment compared to control (Table 4). The T7 and T8 treatments had increased spore density than other treatments. However, there was no significant difference between T7 and T8. The T3 and T4 had higher spore density compared to T1, T2, T5 and T6. The root colonization was significantly (*P* < 0.01) enhanced by the presence of *G. mosseae* spores (Table 4). The root colonization by *G. mosseae* significantly increased in T7 and T8 compared to the other treatments. However, T7 and T8 had no significant difference between them. The root colonization was higher in T3 and T4 than T1, T2, T5 and T6.. However, there was no significant difference between T3 and T4. In rhizosphere, *Enterobacter* sp. population was observed in T5, T6, T7 and T8 treatments at 5.9 x 10^7^ CFU g^-1^ soil, 6.3 x 10^7^ CFU g^-1^ soil, 1.2 x 10^8^ CFU g^-1^ soil and 1.4 x 10^8^ CFU g^-1^ soil, respectively. However, T7 and T8 had no significant difference between them, while both these had significantly higher population of *Enterobacter* sp. than T5 and T6 treatments (Table 4).

**Table 4:** Spore density and root colonization of *G. mosseae* and *Enterobacter* sp. population affected by bioformulation in a glasshouse assay 24 weeks after inoculation.

Bioformulation treatment	No. of AMF spores (per 10 g soil)	AMF root colonization (%)	*Enterobacter* sp. population (CFU g^-1^ soil)
T1 (Control)	5 ± 0.58 c	5 ± 0.81 c	Nd
T2 (Si)	6 ± 0.31 c	4 ± 0.81 c	Nd
T3 (AMF)	54 ± 3.54 b	40 ± 1.63 b	Nd
T4 (AMF + Si)	55 ± 2.89 b	42 ± 1.69 b	Nd
T5 (Eb)	7 ± 1.15 c	6 ± 1.05 c	5.9 × 10^7^ b
T6 (Eb + Si)	7 ± 1.56 c	6 ± 1.24 c	6.3 × 10^7^ b
T7 (Split AMF + Eb + Si)	72 ± 4.42 a	52 ± 2.70 a	1.2 × 10^8^ a
T8 (Consortium of Eb + AMF + Si)	78 ± 3.67 a	53 ± 2.10 a	1.4 × 10^8^ a

## 4. Discussion

The efficacy of bioformulations is a critical factor during the storage [52]. In our study, over 24 weeks of storage under laboratory conditions, bioformulation of co-inoculants with Si exhibited the highest population density of *Enterobacter* sp. and AMF spores. This outcome may be attributed to the synergistic interaction between these microbial inoculants. The *Enterobacter* genus isolated from different host plants, is considered to be one of the mycorrhizae helper bacteria [53]. The formulation of the *Enterobacter cloacae* HFZ-H4 strain showed better survival, with its population remaining within the permissible limit of 6 x 10^8^ cfu g^**-1**^ at the end of 6 months of storage [54]. Additionally, AMF inoculum could be stored in a vermiculite-based formulation for five months at a temperature range of 20 °C to 30 ºC while maintaining a maximum AMF spore count [35].

Our results from the glasshouse study suggested an overall capacity of peat-based bioformulation of both microbial inoculants, inoculated either alone or the combination of *Enterobacter* sp. and *G. mosseae* inoculum with Si, to positively affect rubber plant growth and nutrient uptake. The results revealed that 24 weeks after treatment, rubber plants treated with bioformulation of microbial inoculants with Si significantly enhanced plant growth performance than bioformulation of single inoculants with Si and control. However, the results indicated that the bioformulation of co-inoculants with Si was not significantly different from the bioformulation of split inoculation of both inoculants, AMF and *Enterobacter* sp., with Si for all measured growth parameters. The observed growth improvement may be attributed to the growth-promoting properties of microbial inoculants incorporated into the formulation. [45]. *Enterobacter* species exhibit diverse growth-promoting activities, including enhancing nutrient uptake, nitrogen fixation, solubilizing inorganic phosphate, and synthesizing antimicrobial compounds. Additionally, they protect plants against phytopathogens [55]. Similarly, AMF contributes to plant growth by increasing nutrient and water uptake [56]. Bioformulations such as these offer a promising alternative to chemical fertilizers by improving plant growth and soil health in a sustainable manner. Studies have shown that AMF inoculation enhances phosphorus and nitrogen availability in plants, reducing the dependency on synthetic fertilizers. For instance, co-inoculation of AMF (*Glomus aggregatum*) with beneficial bacteria has been reported to significantly enhance nutrient uptake and biomass accumulation in sweet basil plants [57].

The synergistic effects between the two microbial groups may depend on the bacteria’s ability to improve the nutrient contents mediated by AMF. Results from the glasshouse study indicated that bioformulation of co-inoculants with Si significantly improved nutrient contents in rubber plants than bioformulation of *Enterobacter* sp. with Si, bioformulation of AMF with Si, and control treatment. This aligns with a previous study showing that the co-inoculation of rhizobacteria and *G. mosseae* using a peat moss-based bacterial inoculum significantly improved P and K assimilation in maize [44]. Additionally, the combined inoculation of AMF (*G. intraradices*) with rhizobia (*Rhizobium tropici* CIAT899) resulted in higher accumulation of N and P compared to a single inoculum in common bean plants [58]. Furthermore, a consortium of PGPR-rhizobia-AMF increased the plant dry weight and nutrient contents of wheat and faba bean than single microbial inoculation and the control [59].

The results also indicated that *Enterobacter* sp. population in the rhizosphere of plants inoculated with the bioformulation of a consortium of inoculants with Si was significantly higher than in those inoculated with the bioformulation of *Enterobacter* sp. with Si alone. In the glasshouse experiment, results further showed that the *G. mosseae* spore density, as well as root colonization, were significantly increased when plants were treated with bioformulation of a consortium of inoculants with Si compared to bioformulation of AMF with Si and treatments without added AMF treatments. This result may be attributed to the ability of *Enterobacter* sp. to survive and proliferate more effectively in the rhizosphere in the presence of *G. mosseae* within the bioformulation. It has been demonstrated that a peat based formulation of *P. fluorescens* maintained a population of 19.5 x 10^7^ cfu g^-1^ soil in the rhizosphere of maize [60]. Additionally, the combined inoculation of microbial agents (*Penicillium oxalicum*, *Bacillius subtilis* and *Trichoderma harzianum*) and AMF (*Glomus mosseae*) increased both root colonization and bacterial population in rhizosphere soil [61]. A previous study also revealed that co-inoculation of AMF and rhizobacteria using peat moss-based inoculum significantly increased the root colonization compared to control treatment in maize plants [44].

## 5. Conclusions

Our findings demonstrate that a peat moss-based formulation of *Enterobacter* sp. UPMSSB7, *G. mosseae*, and Si effectively sustained the high viability of both inoculants over 24 weeks of storage while significantly enhancing plant growth compared to single-inoculant bioformulations and the control. This bioformulation could be an effective approach to promoting rubber plant growth at the nursery stage. Further investigation of the tested bioformulation is recommended to assess its potential for enhancing rubber plant growth in the field. This study highlights the importance of peat moss-based bioformulations in improving the growth of rubber seedlings. This approach may contribute to the development of a new generation of biofertilizers for sustainable rubber crop production.

## Supporting information

S1 Fig The effects of bioformulation treatments on growth performance of rubber plants (PB-350) compared to control treatment 24 weeks after inoculation. Note: T1 (Control): sterile peat was applied; T2 (Si): sterile peat containing Si (4 g) was applied; T3 (AMF): peat-based bioformulation containing AMF was applied; T4 (AMF + Si): peat-based bioformulation containing AMF with Si (4 g) was applied; T5 (Eb): formulation containing *Enterobacter* sp. was applied; T6 (Eb + Si): formulation containing *Enterobacter* sp. with Si was applied; T7 (Split AMF + Eb + Si): formulation of AMF alone was applied initially at start and then, a week later *Enterobacter* sp. and Si were applied; T8 (Consortium of AMF + Eb + Si): formulation of *Enterobacter* sp., AMF and Si, were applied altogether.
(TIF)

S1 Table (XLSX)
