# Supplementary figures and images for: Efficacy of peat-based bioformulation of microbial co-inoculants with silicon for growth promotion of rubber plants
Source: PLoS One. 2025 Oct 8;20(10):e0331899. doi: 10.1371/journal.pone.0331899 (PMC12507292; doi:10.1371/journal.pone.0331899)

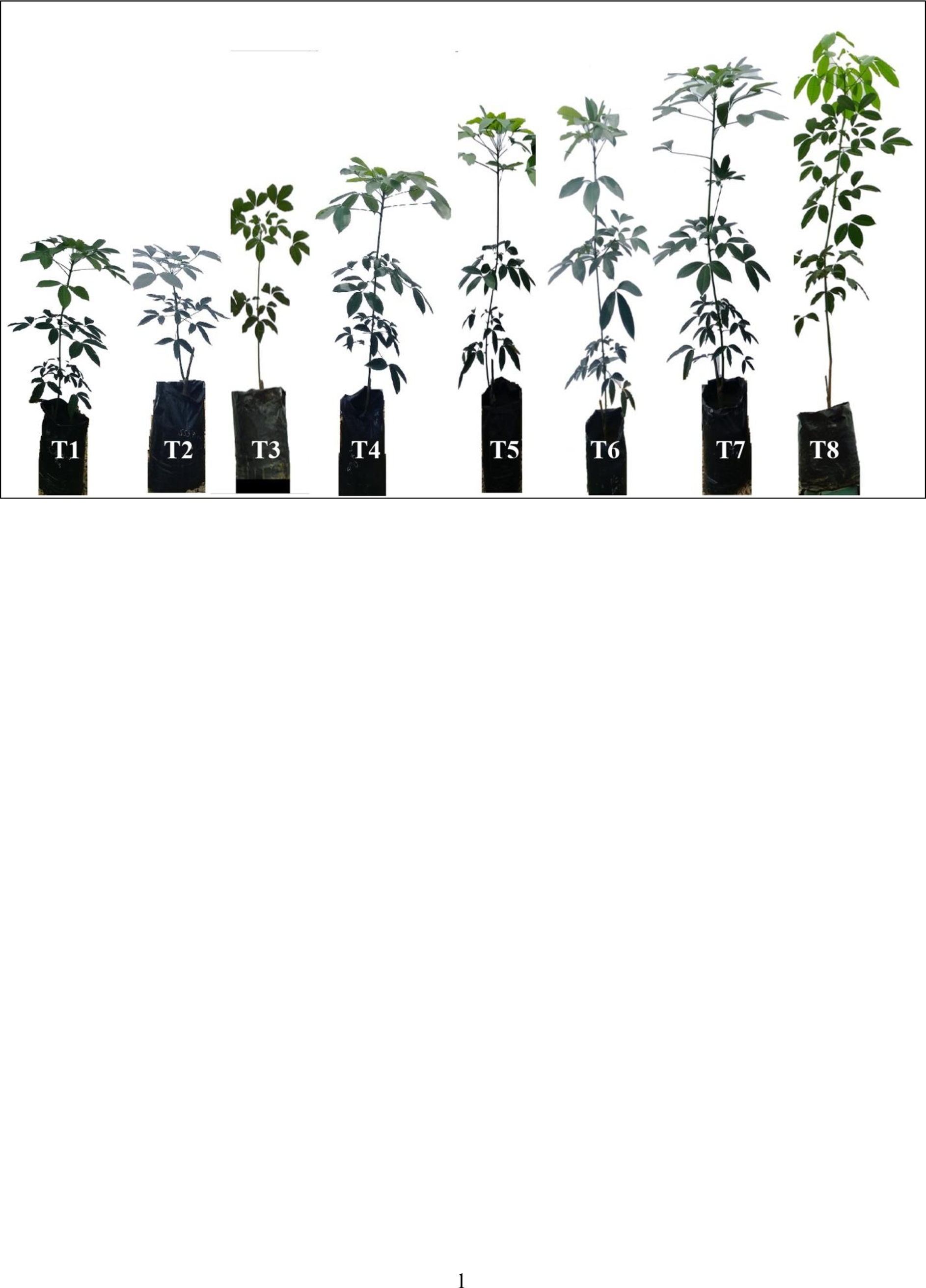

Supplement: S1 Fig — Note: T1 (Control): sterile peat was applied; T2 (Si): sterile peat containing Si (4 g) was applied; T3 (AMF): peat-based bioformulation containing AMF was applied; T4 (AMF + Si): peat-based bioformulation containing AMF with Si (4 g) was applied; T5 (Eb): formulation containing Enterobacter sp. was applied; T6 (Eb + Si): formulation containing Enterobacter sp. with Si was applied; T7 (Split AMF + Eb + Si): formulation of AMF alone was applied initially at start and then, a week later Enterobacter sp. and Si were applied; T8 (Consortium of AMF + Eb + Si): formulation of Enterobacter sp., AMF and Si, were applied altogether. (TIF) [file pone.0331899.s001.tif]
